# Development of a data platform for monitoring personal health records in Japan: The Sustaining Health by Integrating Next-generation Ecosystems (SHINE) Study

**DOI:** 10.1371/journal.pone.0281512

**Published:** 2023-02-14

**Authors:** Haruhisa Fukuda, Fumiko Murata, Sachie Azuma, Masahiro Fujimoto, Shoma Kudo, Yoshiyuki Kobayashi, Kenshi Saho, Kazumi Nakahara, Rei Ono

**Affiliations:** 1 Department of Health Care Administration and Management, Kyushu University Graduate School of Medical Sciences, Fukuoka, Japan; 2 Center for Cohort Studies, Kyushu University Graduate School of Medical Sciences, Fukuoka, Japan; 3 Human Augmentation Research Center, National Institute of Advanced Industrial Science and Technology (AIST), Chiba, Japan; 4 Department of Intelligent Robotics, Toyama Prefectural University School of Engineering, Toyama, Japan; 5 Department of Rehabilitation, Faculty of Health Sciences, Kumamoto Health Science University, Kumamoto, Japan; 6 Department of Public Health, Kobe University Graduate School of Health Sciences, Hyogo, Japan; Duervation, AUSTRIA

## Abstract

**Background:**

The Sustaining Health by Integrating Next-generation Ecosystems (SHINE) Study was developed as a data platform that incorporates personal health records (PHRs) into health-related data at the municipal level in Japan. This platform allows analyses of the associations between PHRs and future health statuses, and supports the production of evidence for developing preventive care interventions. Herein, we introduce the SHINE Study’s profile and describe its use in preliminary analyses.

**Methods:**

The SHINE Study involves the collection of participants’ health measurements and their addition to various health-related data from the Longevity Improvement & Fair Evidence (LIFE) Study. With cooperation from municipal governments, measurements can be acquired from persons enrolled in government-led long-term care prevention classes and health checkups who consent to participate in the SHINE Study. For preliminary analyses, we collected salivary test measurements, lifelog measurements, and gait measurements; these were linked with the LIFE Study’s database. We analyzed the correlations between these measurements and the previous year’s health care expenditures.

**Results:**

We successfully linked PHR data of 33 participants for salivary test measurements, 44 participants for lifelog measurements, and 32 participants for gait measurements. Only mean torso speed in the gait measurements was significantly correlated with health care expenditures (*r* = -0.387, *P* = 0.029).

**Conclusion:**

The SHINE Study was developed as a data platform to collect and link PHRs with the LIFE Study’s database. The analyses undertaken with this platform are expected to contribute to the development of preventive care tools and promote health in Japan.

## Introduction

Japan is the world’s most aged country, with almost 30% of its population aged ≥65 years in 2020 [1]. For many years, Japan’s universal health system has provided affordable high-quality care to its population with equity and efficiency [2]. However, economic stagnation and population decline have substantially increased the share of gross domestic product spent on health care [3]. Furthermore, insurers are facing an impending financial crisis in which health insurance societies that cover younger workers are disproportionately supporting government health insurance programs in municipalities with high concentrations of older persons [2], thereby placing an unsustainable strain on the insurance system’s financial structure. Japan is also dealing with major national issues, such as a lackluster economy and rapid demographic transition, which have increased the burden on public funds. Therefore, in addition to the conventional approach of treating a disease after onset, there is a pressing need to introduce and strengthen systems for preemptive interventions to prevent disease occurrence and reduce their clinical and economic burdens.

Within Japan’s health care infrastructure, municipalities could play a key role in establishing a system that can identify at-risk persons within a population and provide targeted preventive care. This is because municipal governments serve as insurers for National Health Insurance enrollees, which include retirees aged 65–74 years as well as self-employed persons and primary industry workers regardless of age. In addition, municipal governments also serve as insurers for long-term care (LTC), and are responsible for providing preventive care with the aim of reducing the growing demand for LTC services. In their role as insurer, each municipal government routinely acquires and stores a wide variety of resident data, including health care claims data, LTC claims data, health checkup data, LTC prevention class enrollee data, residence data, and income data. Despite possessing an abundance of data to screen for persons who require preemptive interventions, municipal governments are currently unable to do so for several reasons. First, there are no common resident-specific ID numbers or codes across the different data types, which prevents data linkages. Second, the Kokuho Database (KDB) System used by municipal governments only allows insurance claims data to be stored for 5 years, and therefore cannot support long-term follow-up analyses. Third, municipal government workforces do not usually include specialists (e.g., epidemiologists and data scientists) who can conduct rigorous screenings and analyses. Finally, municipal governments do not collect and store residents’ personal health records (PHRs) that are not essential for government administration, even if such records are needed to identify at-risk persons.

By introducing an early screening and preventive care intervention system, we may be able to ascertain the warning signs of a disease and predict its onset in individual residents, notify them of the risks, provide guidance on preventive measures, and prevent or delay disease occurrence. The prevention and delay of acute diseases that require high-cost care could also alleviate the need for LTC services. In this way, the widespread implementation of targeted preemptive interventions could extend the healthy lifespan of Japan’s population, reduce lifetime medical expenditures, and contribute to the control of rising health care costs. To aid the provision of preventive care, we developed the Sustaining Health by Integrating Next-generation Ecosystems (SHINE) Study, which adds PHRs to routinely collected data by municipal governments to support research. Herein, we introduce the SHINE Study’s profile and describe its use in several preliminary analyses.

## Methods

### SHINE Study overview

**Fig 1:**
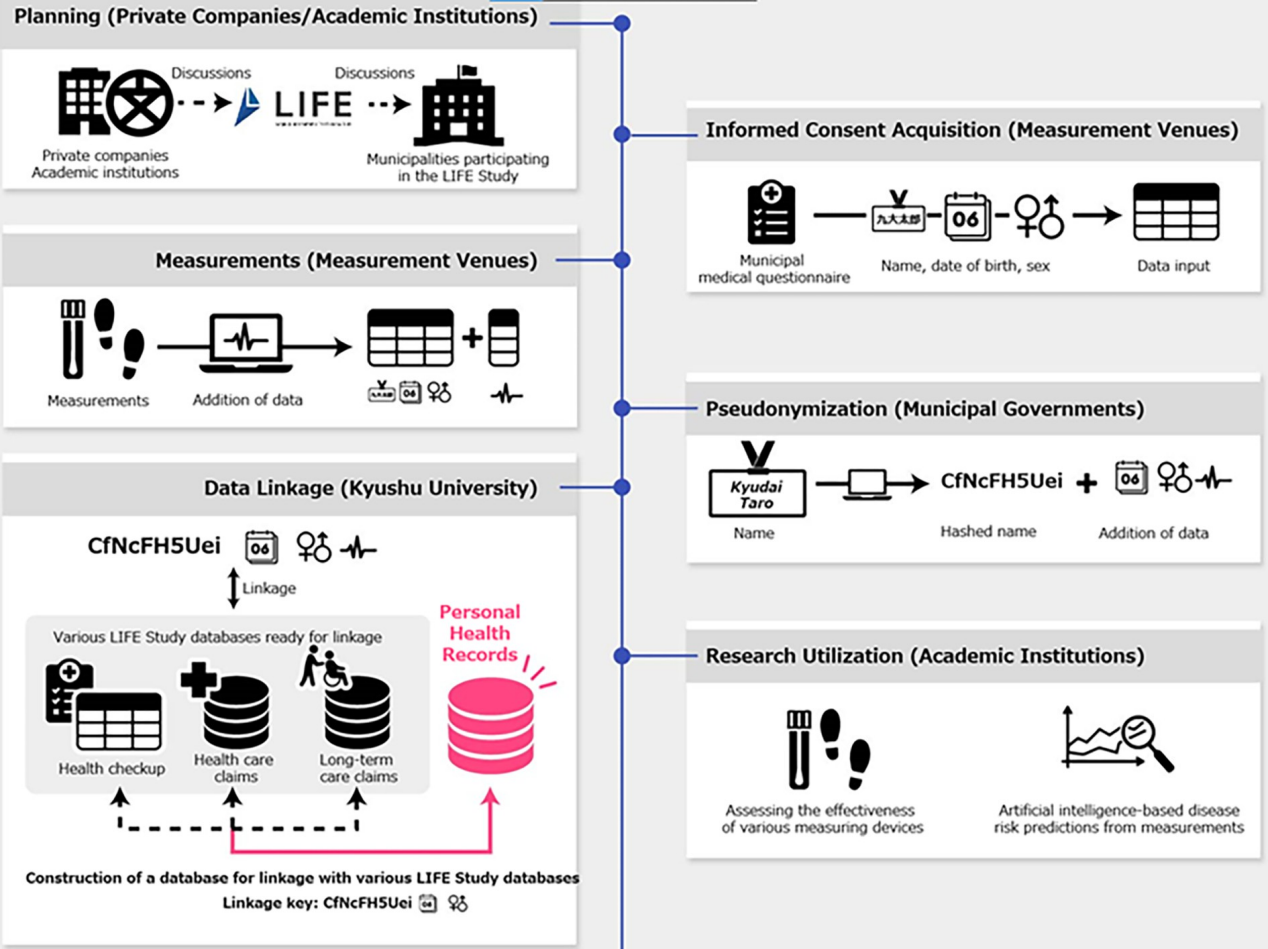
Flowchart of database construction in the SHINE Study.

The SHINE Study is a data platform designed to support cohort studies on the associations between various health measurements and health statuses. Specifically, the platform incorporates PHRs into a database developed and maintained by the Longevity Improvement & Fair Evidence (LIFE) Study in Kyushu University (Fukuoka, Japan). While details are provided elsewhere [4], the LIFE Study is a database project in which participating municipalities provide pseudonymized health care claims data, LTC claims data, health data, and government administrative data to Kyushu University, which links these data types at the individual resident level. Under Japan’s Act on the Protection of Personal Information of 2003, “anonymously processed information” refers to personal information relating to a specific individual that is processed such that the individual can neither be identified through certain prescribed actions nor can the information be restored. The LIFE Study assigns unique research ID codes to individuals after their personally identifiable information are removed. However, each participating municipal government maintains a correspondence table that links these research ID codes with their matching residents’ names, which allows for the later addition of data items. While LIFE Study staff and researchers have no access to these correspondence tables, the use of research ID codes means that these data were pseudonymized rather than anonymized. The resulting multi-source database facilitates long-term follow-up analyses of residents. However, the data types used by the LIFE Study are limited to those collected solely for the purpose of administrative procedures, and therefore lack PHRs that could provide insight into the clinical need for preventive care. To overcome this shortcoming, the SHINE Study was developed (with cooperation from LIFE Study participating municipalities) in 2021 as a semi-open data platform characterized by the following 3 features: (1) Collection of PHRs from individuals enrolled in LTC prevention classes and health checkups conducted by municipal governments, (2) linkage of these PHRs with the LIFE Study database, and (3) construction and validation of disease prediction models based on these PHRs. The SHINE Study aims to create new data ecosystems that can be used by private companies and academic institutions with approval from the participating municipalities (**Fig 1**).

In the first feature described above, participants’ health-related markers are measured by evaluators using sensor technologies owned by private companies and academic institutions, and these measurements are collected as PHRs. The LIFE Study coordinates discussions between the participating municipalities and interested companies/institutions (“Planning” phase in **Fig 1**). For each series of measurements, the SHINE Study obtains approval from the municipalities and LTC prevention class/health checkup administrators. Therefore, this platform can be considered to be semi-open. For the preliminary analyses, we examined commercial salivary diagnostic test measurements, lifelog measurements, and gait measurements. In the SHINE Study, lifelog refers to an individual’s daily activities (e.g., step count, walking distance, and calories burned) recorded as digital data using wearable or mobile devices. The acquisition and analysis of these PHRs was approved by the Kyushu University Institutional Review Board for Clinical Research (Approval Number: 20212002).

In the second feature, the PHRs are linked with the LIFE Study database under strict privacy protection measures. First, written informed consent is acquired from each participant at the measurement venues (“Informed Consent Acquisition” phase in **Fig 1**), and the participants’ measurements are recorded (“Measurements” phase in **Fig 1**). Next, the recorded measurements are provided to the municipal governments, which convert the participants’ names to hash values (“Pseudonymization” phase in **Fig 1**). The hashed names and recorded measurements are then sent to Kyushu University. As the participating municipalities already periodically provide LIFE Study data (including hashed names as linkage keys) to Kyushu University, the PHRs can be linked with the existing LIFE Study database (“Data Linkage” phase in **Fig 1**). Subsequently, the data can be used in research (“Research Utilization” phase in **Fig 1**).

**Fig 2:**
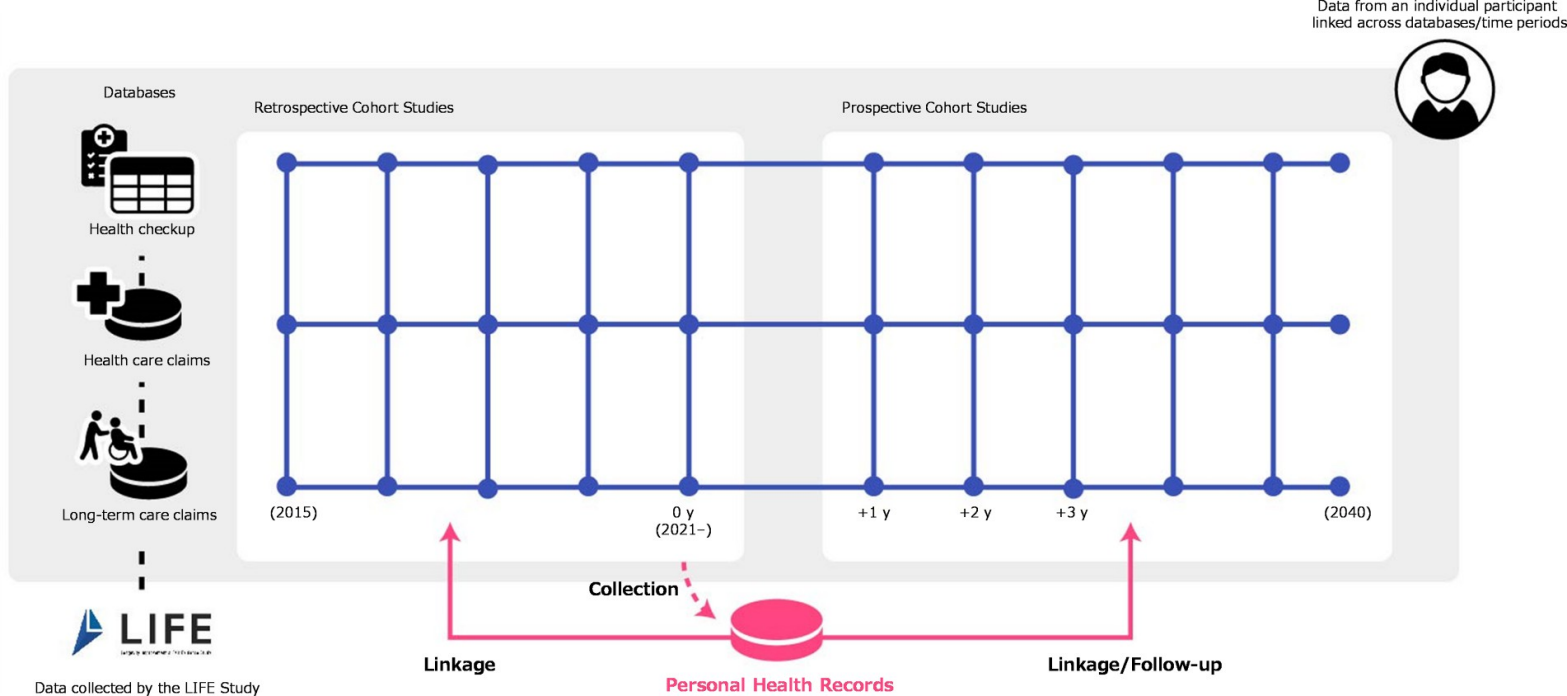
SHINE Study data platform.

In the third feature, disease prediction models are developed using the health care claims data, LTC claims data, health checkup data, and other data (e.g., various screening results, health services participation, certified LTC needs status, income, and residence) that are scheduled to be collected and updated every year until 2040. Therefore, by incorporating PHRs into the LIFE Study database, the health statuses of these individuals can be followed-up over time (**Fig 2**). This enables the development of disease prediction models through prospective cohort studies that assess the associations between the PHRs and the later occurrence of diseases. Eventually, these models could be used to predict the future health statuses of participants immediately after measurements, and may have applications in the targeted implementation of early preemptive interventions. Moreover, this platform can support the linkage of PHRs with previous LIFE Study data, thereby allowing retrospective cohort studies to explore the associations between the participants’ past health statuses and their current PHRs. In this way, the SHINE Study provides a platform that can support analyses using both retrospective and prospective cohort designs.

### SHINE Study preliminary analyses

Using the platform constructed by the SHINE Study, we produced PHRs, linked these records with LIFE Study data, and conducted preliminary analyses using retrospective cohorts. These studies were conducted on salivary diagnostic test measurements, lifelog measurements, and gait measurements. All measurements were taken with approval and cooperation from the participating municipalities. In addition, written informed consent was obtained from each participant before measurement.

As our measurements were taken recently in 2021 and 2022, we are as yet unable to explore their associations with later disease occurrences. Therefore, we retrospectively examined the correlations between each measurement item and the log-transformed health care expenditures in the previous fiscal year (obtained from the health care claims data). *P* values below 0.05 were considered statistically significant.

#### Salivary diagnostic test measurements

Salivary diagnostic tests were performed using the commercially available multi-item rapid Salivary Multi Test kit (LION Dental Products Co., Ltd., Tokyo, Japan). Salivary samples were collected on test strips using mouthwash, and the following levels were measured using a score of 1–100: cariogenic bacteria, pH, buffering capacity, occult blood, leukocytes, protein, and ammonia. This test allows evaluations of the participant’s dental health, periodontal health, and oral hygiene. In addition, each participant was asked to answer a questionnaire on the following items before measurement: received any dental treatment within the past 3 days, use of antibiotics, tendency to choke, consumption of food within the past 2 hours, frequency of brushing, and dental checkups.

#### Lifelog measurements

For the lifelog measurements, participants who agreed to take part in the study were loaned wearable lifelogging devices. In this study, the participants used the Fitbit Versa 3 smartwatch (Fitbit Japan, Tokyo, Japan). The participants were asked to wear the smartwatch during their everyday lives for approximately 1 month. Each participant’s height and weight were recorded into the device at the start of the monitoring period. The lifelog measurements included step count, walking distance, calories burned, calories burned through activity, number of stairs climbed, sleeping duration, and resting heart rate.

#### Gait measurements

Participants who agreed to take part in the study were asked to walk independently at a self-selected pace over a distance of 10 meters, and their locomotion data were recorded using a Doppler radar and accelerometer [5, 6]. The Doppler radar used was a 24 GHz continuous wave radar (Gait Measurement Doppler Sensor, Nitteku Media K.K., Tokyo, Japan). The accelerometers used were an accelerator sensor (Microstone Corporation, Nagano, Japan) and the Human Objective Locomotion MEasurement System (HOLMES) developed by the National Institute of Advanced Industrial Science and Technology (Ibaraki, Japan). HOLMES measures the degree of lumbar acceleration during walking, and the measurements can be compared with records in the National Institute of Advanced Industrial Science and Technology Gait Database. Participants can then receive feedback on their type of gait according to 6 predetermined walking patterns. For this study, mean torso speed and maximum lumbar acceleration were measured. In addition, each participant was asked to answer a questionnaire on the following items before measurement: dominant arm, dominant leg, fall experience within the past year, preexisting conditions, chronic diseases, and use of medication.

## Results

### Salivary diagnostic test measurements

**Fig 3:**
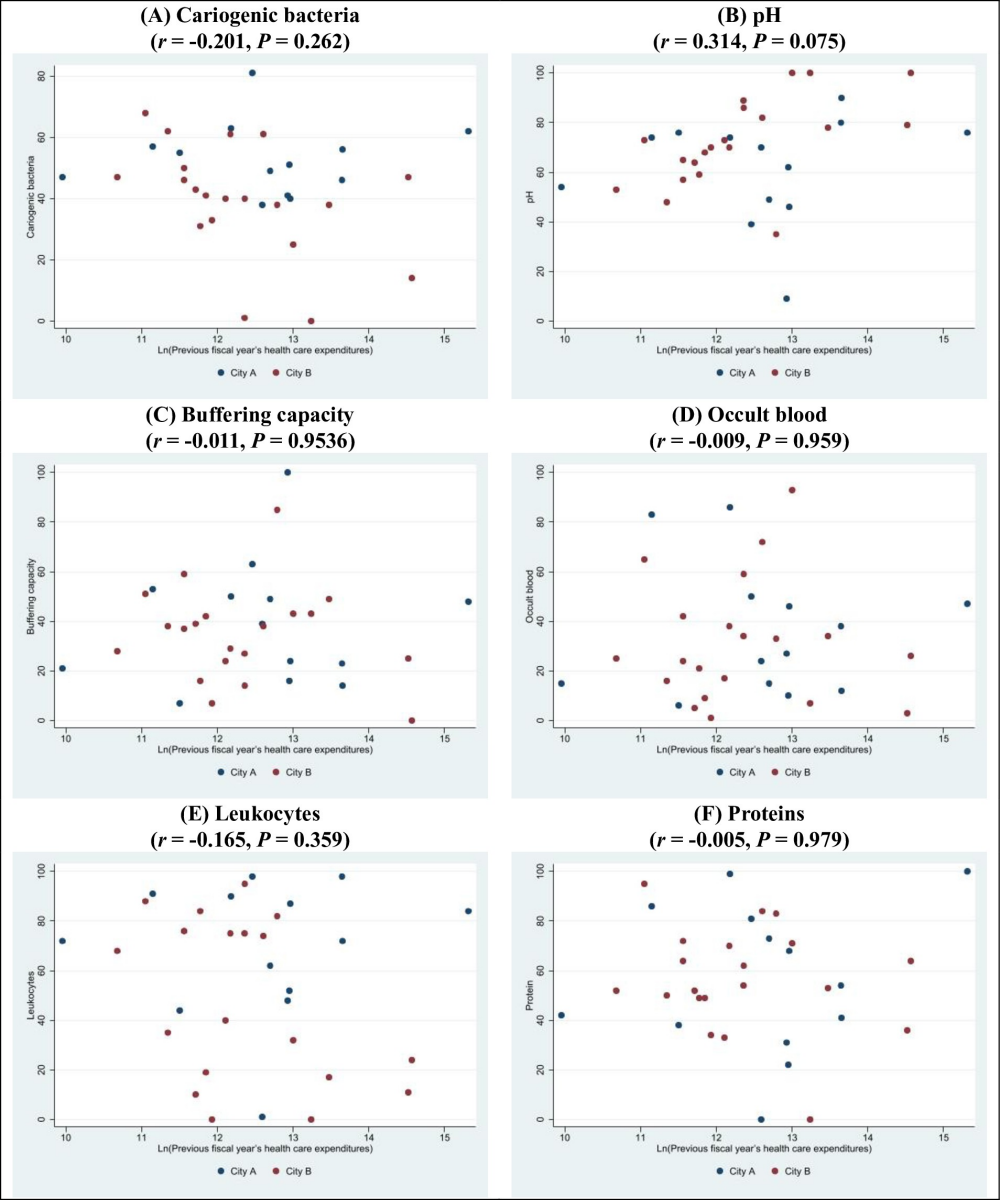
Correlations between salivary diagnostic test measurements and the log-transformed health care expenditures in the previous fiscal year. (A) Cariogenic bacteria (*r* = -0.201, *P* = 0.262); (B) pH (*r* = 0.314, *P* = 0.075); (C) Buffering capacity (*r* = -0.011, *P* = 0.9536); (D) Occult blood (*r* = -0.009, *P* = 0.959); (E) Leukocytes (*r* = -0.165, *P* = 0.359); (F) Proteins (*r* = -0.005, *P* = 0.979).

Salivary diagnostic tests were performed on 39 participants from 2 municipalities (City A and City B). Of these, 6 could not be linked with their health care claims data in the LIFE Study database. Women accounted for the majority of participants in both City A (92.3%) and City B (95.0%). In general, City A had higher cariogenic bacteria and leukocyte counts than City B (**Table 1**). **Fig 3** shows the correlations between the salivary diagnostic test measurements and the log-transformed health care expenditures in the previous fiscal year. None of the measurements showed a significant correlation with these expenditures.

**Table 1:** Salivary diagnostic test measurements.

	City A (n = 13)	City B (n = 20)
Characteristics (Questionnaire)		
Women	12 (92.3%)	19 (95.0%)
Age, years	76.5 [5.4]	78.4 [5.1]
Salivary diagnostic test measurements, score (1–100)		
Cariogenic bacteria	52.8 [11.7]	39.3 [18.6]
pH	61.5 [21.7]	72.5 [17.6]
Buffering capacity	39.0 [25.5]	34.7 [19.1]
Occult blood	35.3 [26.4]	31.2 [24.8]
Leukocytes	69.2 [27.7]	49.1 [33.0]
Protein	56.5 [30.8]	56.4 [21.3]
Ammonia	66.1 [24.0]	55.2 [20.2]
Health care claims data^a^		
Number of comorbidities per person	1.0 [1.0]	1.3 [1.1]
Previous fiscal year’s health care expenditures per person, yen	667,231 [1,168,210]	415,131 [592,065]

Values are presented as number (%) or mean [standard deviation].

^a^ The health care claims data from City A were taken from the year before the month of measurement, and the health care claims data from City B were taken from September 2020 to August 2021.

### Lifelog measurements

**Fig 4:**
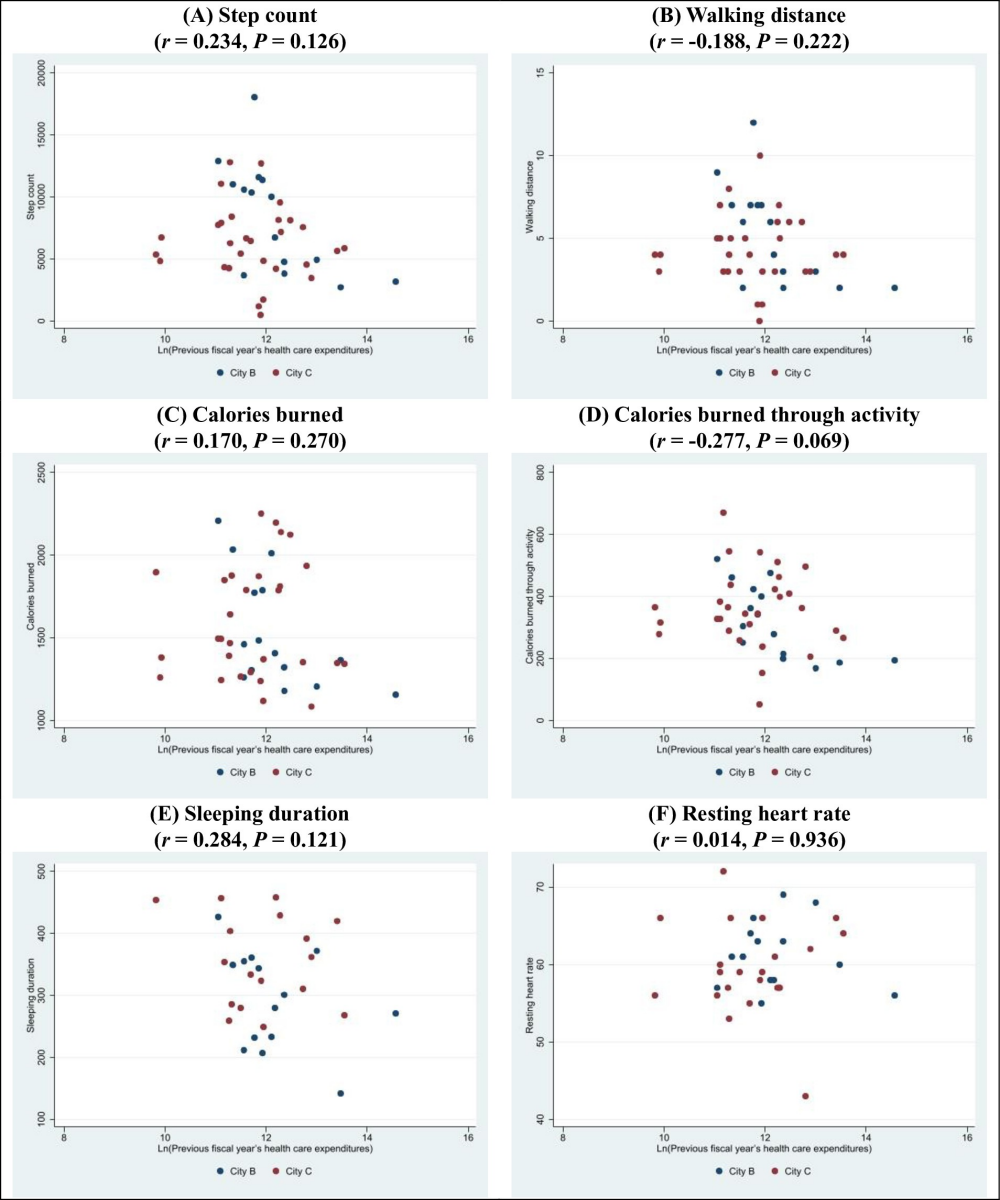
Correlations between lifelog measurements and the log-transformed health care expenditures in the previous fiscal year. (A) Step count (*r* = 0.234, *P* = 0.126); (B) Walking distance (*r* = -0.188, *P* = 0.222); (C) Calories burned (*r* = 0.170, *P* = 0.270); (D) Calories burned through activity (*r* = -0.277, *P* = 0.069); (E) Sleeping duration (*r* = 0.284, *P* = 0.121); (F) Resting heart rate (*r* = 0.014, *P* = 0.936).

Lifelog measurements using the Fitbit smartwatch were performed on 55 participants from 2 municipalities (City B and City C). One participant did not have any measurement data due to a device malfunction, and another participant withdrew from the study prematurely. Nine participants could not be linked with their health care claims data in the LIFE Study database. Women accounted for 93.3% of the participants in City B, but only 55.2% of the participants in City C. The mean step count per day was 8,383 for City B and 6,333 for City C, and the mean sleeping duration per day was 292 minutes for City B and 355 minutes for City C (**Table 2**). **Fig 4** shows the correlations between the lifelog measurements and the log-transformed health care expenditures in the previous fiscal year. Although the results indicated that physical activity was inversely correlated with expenditures, this relationship was not statistically significant.

**Table 2:** Lifelog measurements.

	City B (n = 15)	City C (n = 29)
Characteristics (Questionnaire)		
Women	14 (93.3%)	16 (55.2%)
Age, years	78.7 [5.3]	80.4 [3.0]
Fitbit measurements		
No. of measurement days per person	25.7 [4.8]	30.0 [0]
Step count per day	8,383 [4,484]	6,333 [2,969]
Walking distance per day, km	5.3 [3.0]	4.3 [2.1]
Calories burned per day	1,531 [344]	1,597 [345]
Calories burned through activity per day	319 [117]	358 [127]
Number of stairs climbed per day	4.1 [2.6]	4.8 [2.9]
Sleeping duration per day, mins	292 [80]	355 [73]
Resting heart rate per day, beats/min	61.3 [4.2]	59.5 [5.9]
Health care claims data^a^		
Number of comorbidities per person	0.9 [1.0]	1.1 [1.2]
Previous fiscal year’s health care expenditures per person, yen	334,504 [521,293]	184,491 [178,3]

Values are presented as number (%) or mean [standard deviation].

^a^ The health care claims data from City B were taken from November 2020 to October 2021, and the health care claims data from City C were taken from September 2020 to August 2021.

### Gait measurements

**Fig 5:**
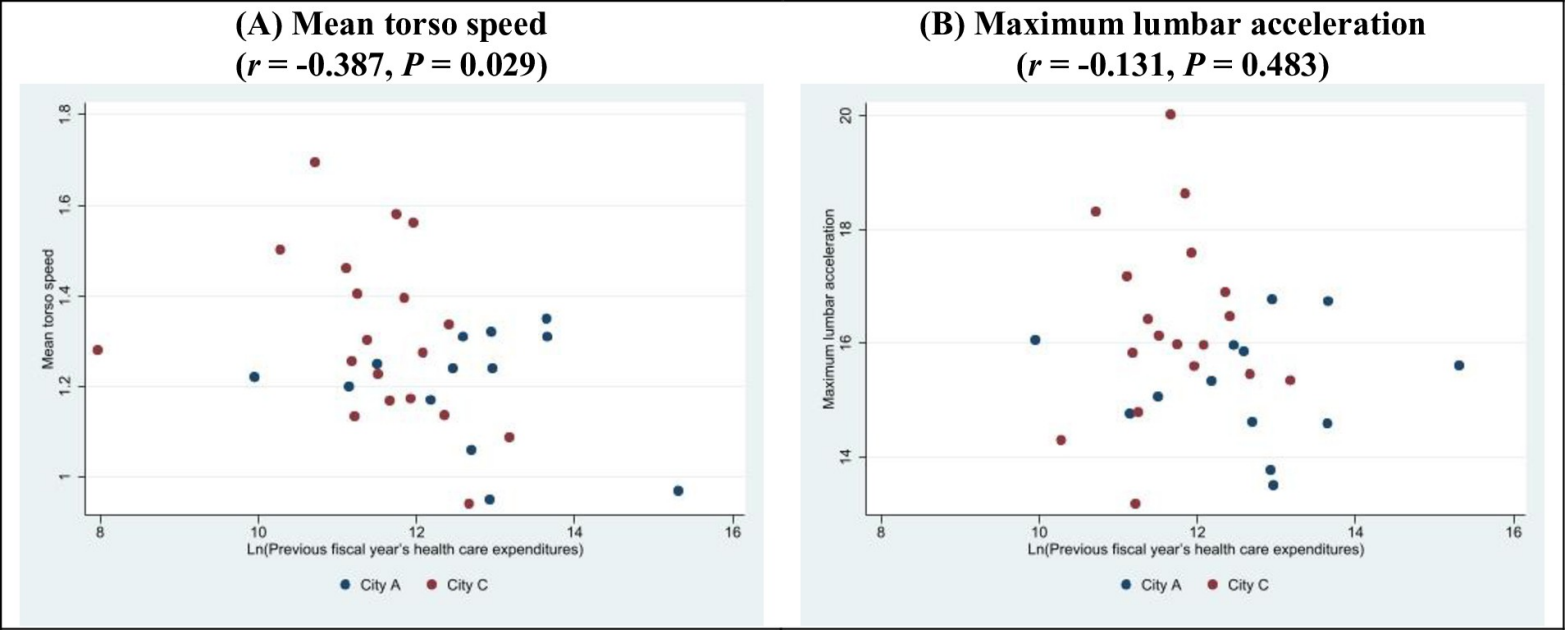
Correlations between gait measurements and the log-transformed health care expenditures in the previous fiscal year. (A) Mean torso speed (*r* = -0.387, *P* = 0.029); (B) Maximum lumbar acceleration (*r* = -0.131, *P* = 0.483).

Gait measurements were performed on 45 participants from 2 municipalities (City A and City C). Two participants walked with the aid of a cane and were excluded from the analysis. Eleven participants could not be linked with their health care claims data in the LIFE Study database. Among the 13 participants in City A, the mean torso speed was 1.2 m/s and maximum lumbar acceleration was 15.3 m/s^2^; among the 19 participants in City C, the mean torso speed was 1.3 m/s and maximum lumbar acceleration was 16.3 m/s^2^ (**Table 3**). **Fig 5** shows the correlations between the gait measurements and the log-transformed health care expenditures in the previous fiscal year. Mean torso speed showed a significant inverse correlation with expenditures (*r* = -0.387, *P* = 0.029), but maximum lumbar acceleration had no such correlation (*r* = -0.131, *P* = 0.483).

**Table 3:** Gait measurements.

	City A (n = 13)	City C (n = 19)
Characteristics (Questionnaire)		
Women	12 (92.3%)	17 (89.5%)
Age, years	76.4 [4.6]	82.4 [3.1]
Gait measurements		
Mean torso speed, m/s	1.2 [0.1]	1.3 [0.2]
Maximum lumbar acceleration, m/s^2^	15.3 [1.0]	16.3 [1.6]
Health care claims data^a^		
Number of comorbidities per person	1.0 [1.0]	0.9 [1.1]
Previous fiscal year’s health care expenditures per person, yen	667,231 [1,168,210]	144,277 [121,566]

Values are presented as number (%) or mean [standard deviation].

^a^ The health care claims data from City A were taken from the year before the month of measurement, and the health care claims data from City C were taken from September 2020 to August 2021.

## Discussion

In this paper, we described the development of the SHINE Study—a data platform that facilitates the collection of PHRs and their linkage with existing health-related and other data. This supports analyses of the associations between PHRs and future health statuses, as well as the construction of disease prediction models at the municipal level in Japan. Using this platform, we conducted preliminary analyses on salivary diagnostic test measurements, lifelog measurements, and gait measurements. We also confirmed the feasibility of linking these PHRs with existing health care and LTC claims data collected by municipal governments. The SHINE Study has potential applications as a verification platform to assess the associations between health measurements taken with various sensor devices and subsequent disease onset.

The SHINE Study platform can strongly support the production of evidence for the implementation of preemptive interventions. For example, in order to assess the associations between physical activity levels (measured using Fitbit or similar devices) and future health outcomes, researchers must usually conduct prospective cohort studies that continuously monitor the participants’ health statuses over medium- to long-term periods. However, such prospective cohort studies generally incur high follow-up costs, are susceptible to participant dropouts, and have limited health-related items for assessments. As a result, studies tend to assess the associations between physical activity levels and current health statuses [7–9]. Although recent studies have developed digital health platforms that enable the longitudinal acquisition of PHRs on smartphones and other devices, these have mostly relied on self-reported results and the measurements do not support integration with other data types from health care providers [10–13]. In the SHINE Study platform, evaluators quantitatively measure the participants’ health-related items using various devices, and incorporate these PHRs into a longitudinal database containing claims data from the LIFE Study for long-term monitoring. Furthermore, our platform is characterized by low participant dropout rates, negligible follow-up costs, and access to all data items available in the claims data. Private companies are also encouraged to participate and use their diverse sensor technologies to produce a wide variety of PHRs. This accumulation of accurate and multidimensional datasets can contribute to modeling the trajectory of disease development over time, and support the implementation of preventive care interventions.

In Japan, the Iwaki Health Promotion Project is a pioneering study on the use of PHRs [14]. In that project, researchers developed a platform-centered system with a wide variety of PHRs that allow analyses of the interrelationships between measured items. Although the SHINE Study has yet to compile a large number of measurement items, our platform is strengthened by the ability to perform data linkages for individual participants with the extensive data items in health care and LTC claims data (including disease names, prescriptions, and procedures) as well as the ability to support medium- to long-term prognostic evaluations. Further participation by private companies and academic institutions will help to expand the array of measurements for this data linkage platform. Numerous region-based cohort studies conducted in Japan have used PHRs to build high-quality databases that allow for long-term follow-up to monitor disease risk in the target populations [15–22]. However, those studies are likely to incur high follow-up costs, and may be overly dependent on the availability of research funds. By using the claims data available in the LIFE Study, the SHINE Study has access to a wide variety of longitudinal data on health statuses without the need for constant funding.

The applications and advantages of the SHINE Study platform should be interpreted with consideration to several limitations. First, the participation of each municipality for each set of measurements is based on individual requests and discussions with the LIFE Study. Therefore, the municipalities may participate only if they deem the measurements to be useful in promoting the health of their residents. Nevertheless, the municipal governments aim to increase continued participation in the LTC prevention classes and health checkups, which could be encouraged by longitudinal measurements with valuable feedback from the SHINE Study. Second, our preliminary analyses used relatively small sample sizes. As these were pilot studies for a variety of measurements, we selected smaller LTC prevention classes comprising approximately 20 enrollees each. In the future, we plan to expand the measurement targets to include larger health checkup venues following approval from the municipal governments. Third, the LIFE Study collects data from enrollees of Japan’s National Health Insurance System and the Latter-Stage Older Persons Health Care System, and older persons may be overrepresented in our study populations. However, the SHINE Study is scheduled to recruit participants from health checkups, which could slightly reduce the representativeness of the sample but include more individuals aged ≥40 years. Fourth, the SHINE Study utilizes health care claims data, LTC claims data, and health data acquired by the LIFE Study in order to track residents’ health statuses. Therefore, residents who are lost to follow-up (due to moving out of a participating municipality, changing insurer, or dying) in the LIFE Study will also be lost to follow-up in the SHINE Study. Fifth, the quality of the PHR data (salivary diagnostic test, lifelog, and gait measurements) has not been validated. Although a systematic review has shown that Fitbit devices have generally acceptable accuracy in step measurements for adults with no mobility limitations [23], verifying the accuracy of these and other PHR measurements remains an important task for the SHINE Study.

## Conclusion

The SHINE Study was developed as a data platform to collect and link PHRs with the LIFE Study’s claims data. With approval and cooperation from the participating municipalities, this project aims to construct scalable data ecosystems that can be used by private companies and academic institutions. The analyses undertaken with the SHINE Study platform are expected to contribute to the development of preventive care tools and promote the health of residents in Japan.
